# Precision Medicine in Acute Coronary Syndromes

**DOI:** 10.3390/jcm13154569

**Published:** 2024-08-05

**Authors:** Andrea Caffè, Francesco Maria Animati, Giulia Iannaccone, Riccardo Rinaldi, Rocco Antonio Montone

**Affiliations:** 1 Department of Cardiovascular and Pulmonary Sciences, Catholic University of the Sacred Heart, 00168 Rome, Italy; andreacaffe97@gmail.com (A.C.); francescoanimati@gmail.com (F.M.A.); rinaldi.riccardo92@gmail.com (R.R.); 2 Department of Cardiovascular Medicine, Fondazione Policlinico Universitario A. Gemelli IRCCS, 00168 Rome, Italy; rocco.montone@gmail.com

**Keywords:** precision medicine, stratified medicine, acute coronary syndrome, MINOCA

## Abstract

Nowadays, current guidelines on acute coronary syndrome (ACS) provide recommendations mainly based on the clinical presentation. However, greater attention is being directed to the specific pathophysiology underlying ACS, considering that plaque destabilization and rupture leading to luminal thrombotic obstruction is not the only pathway involved, albeit the most recognized. In this review, we discuss how intracoronary imaging and biomarkers allow the identification of specific ACS endotypes, leading to the recognition of different prognostic implications, tailored management strategies, and new potential therapeutic targets. Furthermore, different strategies can be applied on a personalized basis regarding antithrombotic therapy, non-culprit lesion revascularization, and microvascular obstruction (MVO). With respect to myocardial infarction with non-obstructive coronary arteries (MINOCA), we will present a precision medicine approach, suggested by current guidelines as the mainstay of the diagnostic process and with relevant therapeutic implications. Moreover, we aim at illustrating the clinical implications of targeted strategies for ACS secondary prevention, which may lower residual risk in selected patients.

## 1. Introduction

Globally, cardiovascular diseases represent the main cause of mortality, with over 9 million deaths estimated to be due to ischemic heart disease (IHD) in 2019 [1]. Among these, acute coronary syndrome (ACS) and sudden cardiac death cause most of IHD-related deaths [2].

Despite significant progress in the understanding of the involved pathophysiological pathways and available technologies, the diagnostic process for individuals with ACS still primarily relies on the 12-lead surface electrocardiogram (ECG), a technology available since 1887 [3,4]. ECG analysis allows quick differentiation between ST-elevation myocardial infarction (STEMI) and non-ST elevation acute coronary syndromes (NSTE-ACS), thus fundamentally contributing to guiding the subsequent therapeutic timing [3]. In clinical practice, myocardial necrosis biomarkers are utilized as cost-effective and non-invasive diagnostic instruments, as their elevation allows the diagnosis of non-ST-elevation myocardial infarction (NSTEMI) among NSTE-ACS patients, leading to an invasive approach [3]. However, neither ECG nor cardiac biomarkers provide information regarding the mechanisms underlying ACS, as it can result from various ischemic triggers, including plaque instability, spontaneous coronary artery dissection (SCAD), coronary spasm, or coronary embolism [5].

Unlike other medical fields, such as oncology or immunology, in which personalized therapies are already used to a great degree, the development of a precision medicine model for ischemic heart disease is still in progress [6,7,8].

Traditionally, ACS has been associated with the destabilization of an atherosclerotic plaque, resulting in the acute thrombotic obstruction of an epicardial coronary artery [9]. For this reason, patients diagnosed with ACS typically receive a relatively standard treatment often involving the administration of dual antiplatelet therapy (DAPT), regardless of the specific underlying pathophysiology, and drug-eluting stent placement in a large extent of cases [3,10]. However, not all cases of MI present with angiographically obstructive coronary artery disease (MI-CAD) [11], since 6–8% of all MI patients present with myocardial infarction with non-obstructive coronary arteries (MINOCA), a heterogeneous clinical entity defined as an ACS occurring in the absence of obstructive coronary artery disease (no coronary artery stenosis ≥ 50% on invasive coronary angiography), requiring a pathophysiology-guided management [5].

Herein, we discuss the role of precision medicine both in patients with MI-CAD and in subjects presenting with MINOCA.

## 2. Precision Medicine in MI-CAD

Recent progress in diagnostic techniques (mainly regarding intravascular imaging and inflammatory biomarkers) demonstrated in vivo that plaque destabilization leading to MI-CAD may be determined by distinct mechanisms, with relevant clinical and prognostic implications [10], confirming data from early pathologic studies. On this premise, the relevance of a precision medicine approach in ACS has been increasingly recognized over recent years, especially regarding culprit and non-culprit lesion assessment [12]. Furthermore, a stratified antithrombotic strategy in MI-CAD represents a crucial component of these patients’ management, particularly if considering individual drug-response variability [13].

### 2.1. Culprit Plaque Assessment

**Figure 1:**
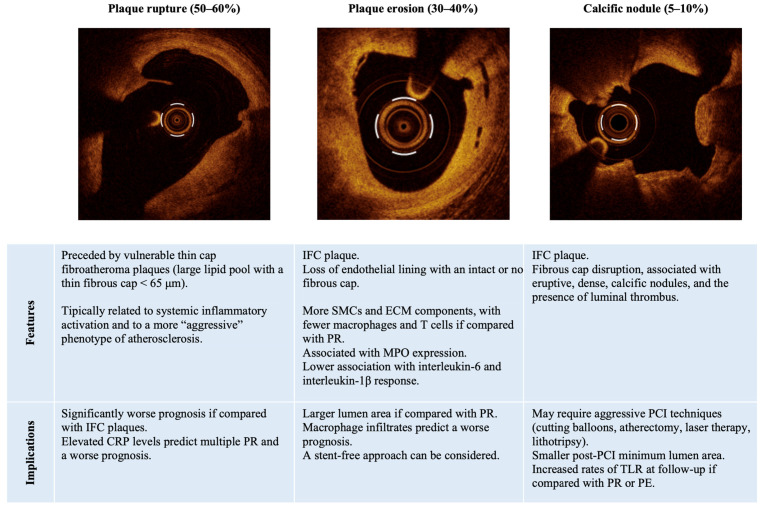
Features and clinical implications of the culprit plaque subtypes associated with MI-CAD. Abbreviations: CPR: C-reactive protein; ECM: extracellular matrix; IFC: intact fibrous cap; MPO: myeloperoxidase; PCI: percutaneous coronary intervention; PE: plaque erosion; PR: plaque rupture; SMCs: smooth muscle cells; TLR: target lesion revascularization.

In patients presenting with ACS, coronary angiography represents a gatekeeper exam, followed by revascularization and stenting of the culprit lesion in most cases [12]. However, a stratified approach can be guided by intravascular imaging, including Optical Coherence Tomography (OCT) and IntraVascular UltraSound (IVUS), which enable cardiologists to differentiate between mechanisms of plaque instability underlying ACS (Figure 1) and allows them to choose the most appropriate therapeutic strategy based on the hypothesized pathophysiological pathway [14].

Considering the morphology of the culprit plaque, the majority of ACS (about 50–60%) are caused by the destabilization of vulnerable thin cap fibroatheroma (TCFA) plaques [10], which are histopathologically defined by a large lipid pool with a thin fibrous cap measuring less than 65 μm, resulting in plaque rupture (PR) [15,16]. TCFA is a notable indicator of plaque progression and a predictor of subsequent adverse clinical outcomes [16]. In addition, a culprit PR portends a significantly worse prognosis if compared with non-ruptured plaques [17]. On the other hand, one third of ACS are caused by non-ruptured (or intact fibrous cap, IFC) plaques, including plaque erosion (PE) and calcified noduli (CN) [18]. PE is histopathologically defined by the loss of the endothelial lining with a non-disrupted fibrous cap [19]. In contrast to a TCFA-related PR, eroded plaques feature smooth muscle cells and extracellular matrix elements such as hyaluronan and proteoglycans, which are found adjacent to the thrombus at the luminal surface, while macrophages and T cells (a hallmark of ruptured plaques) are usually scarce and scattered [19,20]. Eroded plaques typically have a small or undetectable lipid core and tend to have a larger lumen area, with more than half of them exhibiting less than 75% area stenosis [20]. Nevertheless, OCT identification of lipid-rich IFC culprit lesions may allow risk stratification, as it portends major adverse cardiovascular events (MACEs) at follow-up, predominantly due to target vessel revascularization [21].

One of the first advances toward an intracoronary imaging-guided treatment of ACS was provided by the EROSION (Effective anti-thrombotic therapy without stenting: intravascular optical coherence tomography-based management in plaque erosion) study, which suggested that for ACS attributed to PE, stent-free management with low-dose aspirin and ticagrelor might be a safe and effective approach, considering that the majority of individuals experiencing ACS due to PE treated with this strategy (92.5%) were free of MACE for up to one year [22]. The recent EROSION III study investigated whether OCT guidance versus angiographic guidance resulted in fewer stent implantations and improved the prognosis in STEMI patients [23]. In the OCT group, the reperfusion strategy was decided based on the underlying pathogenic mechanism, with a conservative approach adopted in case of PE, PR without dissection or hematoma, or SCAD [23]. The use of OCT led to a 15% reduction in stent implantation rate, which was the primary endpoint of the study [23]. Data coming from a post hoc analysis of the DANAMI-3-DEFER (Deferred versus conventional stent implantation in patients with ST-segment elevation myocardial infarction) trial have shown that STEMI patients with thrombolysis in myocardial infarction (TIMI) flow grade 2–3 and no significant residual stenosis after initial PCI in whom stenting was omitted had a cardiovascular event rate that was comparable to that of patients undergoing standard PCI and immediate stenting [24]. These results are pioneering since they add a piece to the intricate puzzle of stent-free ACS management (Table 1). Furthermore, the abovementioned therapeutic and prognostic implications underscore how intravascular imaging is pivotal to understanding the mechanism underlying ACS, thus tailoring the clinical management of patients.

CN have been identified as the less common culprit plaque subtype in the context of ACS (<10% of cases) [25]. CN plaques are characterized by a lesion with fibrous cap disruption, often associated with eruptive, dense, calcific nodules and the presence of luminal thrombus [25]. CN as mechanisms of ACS have been reported in approximately 4% of all ACS—with a higher prevalence in ostial or mid-right coronary artery [26]. CN can be visualized using both IVUS and OCT imaging techniques. However, OCT offers better detection of thrombus and provides clearer delineation of the superficial and deep boundaries of calcium and plaque disruption, though the attenuation of deeper structures can lead to a misrepresentation of red thrombi and potential misdiagnosis of a PR [12]. Furthermore, according to an OCT study by Kobayashi et al. [27], eruptive CN are linked to a smaller post-percutaneous coronary intervention (PCI) minimum lumen area and to increased rates of target lesion revascularization at follow-up if compared with PR or PE, thus highlighting the complexity of this type of atherosclerotic plaque.

A personalized treatment strategy in ACS patients can also be guided by circulating biomarkers, as they show a clinical significance and different correlations with the type of plaque causing the ACS, with the advantage of representing a non-invasive diagnostic tool [28,29]. High blood levels of C-reactive protein (CRP) has been associated with poorer prognosis in patients with ACS [30] and has shown a positive correlation with multiple PRs, thus suggesting the necessity of a systemic anti-inflammatory approach aimed to lessen the impact of inflammation besides a revascularization-based strategy, possibly tailored on the basis of the individual inflammatory profile [31,32]. On the other hand, systemic levels of myeloperoxidase are significantly higher in patients with ACS who present with culprit PE versus PR [33]. In addition, while PE shows a better prognosis and distinct inflammatory features if compared with PR [34], OCT-assessed macrophage infiltrates at the site of culprit eroded plaques have been associated with features of vulnerability and worse outcome at follow-up [35].

Considering other inflammatory markers, a study by Chandran et al. [36] showed a positive correlation between elevated intracoronary levels of Epidermal Growth Factor and Thrombospondin-1 and IFC plaques, while elevated intracoronary interferon-inducible T-cell alpha chemoattractant was associated with PR.

**Table 1:** Main studies evaluating precision medicine diagnostic and therapeutic strategies in both MI-CAD and MINOCA.

Study	Population	Design	Results/Objectives
PE and stent free management			
EROSION (Jia et al., 2016) [37]	60 ACS patients (58 STEMI, 2 NSTE-ACS) with an OCT diagnosis of PE and <70% residual angiographic DS after thrombus aspiration, TIMI flow grade 3, and no progressive chest pain.	Single-arm, uncontrolled, prospective study investigating the feasibility of DAPT with ticagrelor without stenting in patients with ACS due to PE.	At 1-month follow-up: thrombus volume significantly decreased (94.2%) in the 55 patients who completed their 1-month follow-up. 78.3% had >50% reduction of thrombus volume. 2 patients experienced MACE.
EROSION 1-Year Follow-Up (Xing et al., 2017) [22]	53 ACS patients (51 STEMI, 2 NSTE-ACS) with an OCT diagnosis of PE and <70% residual angiographic DS after thrombus aspiration, TIMI flow grade 3, and no progressive chest pain.	Single-arm, uncontrolled, prospective study investigating the feasibility of DAPT with ticagrelor without stenting in patients with ACS due to PE.	At 1-year follow-up: 92.5% of patients remained free of MACE for up to 1 year. Thrombus volume further decreased between 1 month and 1 year.
EROSION 4-Year Outcomes (He et al., 2021) [38]	52 ACS patients (50 STEMI, 2 NSTE-ACS) with an OCT diagnosis of PE and <70% residual angiographic DS after thrombus aspiration, TIMI flow grade 3, and no progressive chest pain.	Single-arm, uncontrolled, prospective study investigating the feasibility of DAPT with ticagrelor without stenting in patients with ACS due to PE.	At 4-year follow-up: 21% of cumulative rate of TLR (not associated with ACS). More effective thrombus dissolution during the first month predicted better long-term follow-up in terms of TLR.
EROSION III (Jia et al., 2022) [23]	226 STEMI patients with <70% residual angiographic DS after thrombus aspiration, and TIMI flow grade 3.	Open-label, prospective, multicenter, randomized, controlled study of OCT vs. angiographic guidance in STEMI.	Significantly lower rate of stent implantation in the OCT guidance guided group, compared to the angiographic guided group (43.8% vs. 58.8%; *p* = 0.024).
DANAMI-3-DEFER trial post hoc analysis (Madsen et al., 2022) [24]	674 STEMI patient from the DANAMI-3-DEFER study (84 randomized to deferred stenting treated with no subsequent stenting; 590 randomized to standard PCI treated with immediate stenting).	Post hoc analysis comparing patients with TIMI flow grade 2–3 after initial PCI and no significant residual stenosis in whom stenting was omitted vs. patients undergoing standard PCI and immediate stenting.	Comparable event rate between stent-free group and standard PCI group (composite of all-cause mortality, recurrent MI, and TVR) (HR 0.87, 95% CI: 0.48–1.60; *p* = 0.66).
PEPSii (NCT04701385) (Wardley et al., ongoing) [32]	80 NSTEMI patients, 40 stable CAD patients (control group).	Prospective observational pilot study to evaluate the feasibility of studying the differences in endothelial cells and neutrophils between NSTEMI patients presenting with PE or PR as assessed by OCT.	Primary outcome measure: apoptotic circulating endothelial cells. Secondary outcome measures: neutrophils, endothelial progenitor cells, biomarker analysis.
MVO assessment			
OxAMI cohort (Fahrni et al., 2017) [39]	261 patients with STEMI undergoing pPCI.	Prospective study evaluating IMR at the time of pPCI can identify patients at low risk of early major cardiac complications after STEMI.	IMR ≤ 40 identified all patients who were free of major cardiac complications.
OxAMI PICSO study (De Maria et al., 2018) [40]	105 patients with STEMI undergoing pPCI.	Prospective study evaluating PICSO effectiveness in reducing infarct size and IMR in STEMI patients with pre-stenting IMR > 40 units.	Compared to controls, the 25 patients treated with PICSO had a lower IMR at 24–48 h (24.8 [18.5–35.9] vs. 45.0 [32.0–51.3], *p* < 0.001) and lower infarct size at six months (26.0% [20.2–30.0] vs. 33.0% [28.0–37.0], *p* = 0.006).
PiCSO-AMI-I Trial (De Maria et al., 2024) [41]	145 patients with STEMI undergoing pPCI.	Prospective, randomized trial evaluating PICSO effectiveness in reducing infarct size, MVO occurrence, and intramyocardial hemorrhage in STEMI patients.	No differences were observed in infarct size, nor in terms of the occurrence of MVO or intramyocardial hemorrhage.
Guided antiplatelet therapy de-escalation			
ANTARCTIC (Cayla et al., 2016) [42]	877 patients 75 years or older who had undergone coronary stent implantation for ACS.	Multicenter, open-label, blinded-endpoint, randomized controlled superiority trial of prasugrel 5 mg qd with dose or drug adjustment in case of inadequate response assessed by PFT (monitoring group) vs. prasugrel 5 mg qd with no monitoring (conventional group).	PFT did not improve clinical outcomes (composite endpoint of CV death, MI, stroke, stent thrombosis, urgent revascularisation) in the monitoring group, compared with the conventional group (HR 1.003, 95% CI 0.78–1.29; *p* = 0.98) The rate of bleeding events did not significantly differ between groups.
TROPICAL-ACS (Sibbing et al., 2017) [43]	2610 ACS patients treated with PCI with a planned DAPT duration of 12 months.	Randomized, open-label, assessor-blinded, multicenter trial comparing prasugrel for 12 months (control group) with a PFT-guided step-down regimen to clopidogrel maintenance therapy after 1 week of prasugrel (guided de-escalation group).	At 1 year after PCI, guided de-escalation of antiplatelet treatment was non-inferior to standard treatment with prasugrel in terms of CV death, MI, or stroke (p_non-inferiority_ = 0.0004; HR 0.81, 95% CI 0.62–1.06, p_superiority_ = 0.12). No significant difference in bleeding events was observed (HR 0.82, 95% CI 0.59–1.13; *p* = 0.23).
POPular Genetics (Claassens et al., 2019) [44]	2488 patients undergoing primary PCI with stenting implantation.	Randomized, open-label, assessor-blinded trial comparing the administration of a P2Y12 inhibitor based on early CYP2C19 genetic testing (genotype-guided group) with a standard treatment with either ticagrelor or prasugrel (standard-treatment group) for 12 months.	CYP2C19 genotype-guided strategy (with clopidogrel being assigned to noncarriers of CYP2C19*2 or CYP2C19*3 loss-of-function) was associated with a lower risk of bleeding (HR 0.78, 95% CI 0.61–0.98; *p* = 0.04) and was non-inferior to standard treatment with ticagrelor or prasugrel at 12 months in terms of death from any cause, MI, definite stent thrombosis, or stroke (absolute difference, −0.7%; 95% CI, −2.0 to 0.7; p_non-inferiority_ < 0.001).
Guided antiplatelet therapy escalation			
PHARMCLO (Notarangelo et al., 2018) [45]	888 ACS patients (prematurely stopped: 24.6% of the pre-specified sample size).	Randomization to standard of care or pharmacogenomic approach (including genotyping of ABCB1, CYP2C19*2, and CYP2C19*17).	Significantly lower risk of primary composite endpoint of CV death, MI, stroke, and major bleeding in the pharmacogenomic arm (HR 0.58; 95% CI 0.43–0.78; *p* < 0.001). Ticagrelor use was significantly higher in the pharmacogenomic arm (42.6% vs. 32.7%; *p* = 0.02).
Al-Rubaish et al. (2019) [46]	755 STEMI patients.	Randomization to a genotype-guided approach or to standard treatment.	31 patients carrying a loss-of-function allele in the genotype-guided arm were treated with ticagrelor. All other patients received clopidogrel. Significantly lower risk of CV death, MI, stroke, and major bleeding (OR 0.34; 95% CI 0.20–0.59; *p* = 0.0001). Non-significant difference in stent thrombosis (OR 0.85; 95% CI 0.43–1.71; *p* = 0.65).
TAILOR-PCI (Pereira et al., 2020) [47]	5302 patients undergoing PCI for ACS or stable CAD.	Open-label, randomization to a genotype-guided group (undergoing point-of-care genotyping, CYP2C19 LOF carriers being prescribed ticagrelor, while noncarriers clopidogrel) or to a conventional group (receiving clopidogrel).	At 12 months, no significant difference was observed in a composite endpoint of CV death, MI, stroke, stent thrombosis, and severe recurrent ischemia (HR 0.84; 95% CI 0.65–1.07; *p* = 0.16), nor minor or major bleeding (HR 1.22; 95% CI 0.60–2.51; *p* = 0.58).
MINOCA			
PROMISE (NCT05122780) (Montone et al., ongoing) [48]	145 MINOCA patients.	Randomized, multicenter, prospective, open-label, superiority trial comparing a “precision medicine approach” versus “standard of care approach” in MINOCA patients.	Primary endpoint: angina status evaluated by SAQSS at 12-month follow-up. Secondary endpoints and exploratory analysis: MACE, healthcare cost analysis, CMR characteristics, circulating biomarkers, diagnostic utility of the “precision medicine approach”.
StratMed-MINOCA (NCT05198791) (Berry et al., ongoing) [49]	300 MINOCA patients.	Prospective, randomized, open-label, endpoint blinded trial comparing eplerone 25–50 mg vs. standard of care in patients with IMR ≥ 25 without heart failure or LVEF ≤ 40%.	Change in NT-proBNP levels at six months. Prospective measurement of coronary physiology parameters including CFR, RRR, and LVEDP.
Inflammation			
CANTOS Trial (Ridker et al., 2017) [50]	10,061 patients with previous MI and a hs-CRP level of <2 mg/L.	Double-blinded, randomized to canakinumab (50 mg, 150 mg, and 300 mg s.c. every 3 months) or placebo.	Canakinumab, at a dose of 150 mg every three months, resulted in a significantly lower rate of recurrent cardiovascular events than placebo (HR 0.83; 95% CI 0.73–0.95; *p* = 0.005).
MRC-ILA Heart Study (Morton et al., 2015) [51]	182 patients with NSTE-ACS within 48 h from symptoms onset.	Double-blinded, randomized to daily IL-1ra s.c. or placebo for 14 days.	Significant reduction of hs-CRP and IL-6 at 14 days in the IL-1ra group. Significant MACE excess at 1 year in the IL-1ra group, driven by a non-significant increase in recurrent MI.
VCUART3 (Abbate et al., 2020) [52]	99 patients with STEMI.	Randomized, double-blind, clinical trial evaluating IL-1ra vs. placebo.	Significant reduction of hs-CRP and improvement of heart failure outcomes in the IL1-ra group.
ASSAIL-MI (Broch et al., 2021) [53]	199 patients with STEMI within 6 h from symptoms onset.	Randomized, double-blind trial evaluating tocilizumab vs. placebo.	Increased myocardial salvage, reduced MVO. No significant difference in infarct size.
LILACS (Zhao et al., 2022) [54]	26 patients with stable CAD. 18 patients with NSTE-ACS.	Randomized, double-blind, dose-escalation trial, testing low-dose s.c. aldesleukin (recombinant IL-2) vs. placebo.	Recombinant low-dose IL-2 expandend regulatory T cells. One serious adverse event after drug administration.
IVORY (NCT04241601) (Sriranjan et al., ongoing) [55]	60 patients with ACS and hs-CRP > 2 mg/L.	Double-blind, randomized, phase II clinical trial testing low-dose s.c. aldesleukin vs. placebo.	Change in mean maximum target to background ratios (TBRmax) in the index vessel as assessed by ^18^F-FDG PET-CT at follow-up. Changes in circulating T-cell subtypes and safety endpoints.
COLCOT (Tardif et al., 2019) [56]	4746 patients with recent MI (within 30 days)	Randomized, double-blinded trial of colchicine vs. placebo.	Significant reduction in the combined endpoint of death from CV causes, resuscitated cardiac arrest, MI, stroke, or urgent hospitalization for angina leading to coronary revascularization (HR 0.77; 95% CI 0.61–0.96; *p* = 0.02). Significantly higher pneumonia incidence in the treated arm (*p* = 0.03).
CLEAR-SYNERGY (NCT03048825) (Sanjit et al., ongoing) [57]	Patients with MI who have undergone PCI. >7000 enrolled patients (primary completion in June 2024).	Randomized, blinded, double-dummy, 2 × 2 factorial design trial of colchicine 0.5 mg vs. placebo and spironolactone 25 mg vs. placebo.	MACE at follow-up.
FLAVOUR (Prescott et al., 2022) [58]	128 MI patients with <50% left anterior descending coronary artery stenosis and TIMI flow grade ≥ 2 after PCI.	Parallel-group trial with 2:1:2 randomization to receive once-daily 5-lipoxygenase-activating protein inhibitor AZD5718 200 mg, AZD5718 50 mg or placebo.	Urine leukotriene E4 levels were significantly reduced (>80%) in the treated arms. No significant changes in non-invasive CFR. No treatment-related serious adverse events.

Abbreviations: ACS: acute coronary syndrome; CAD: coronary artery disease; CFR: coronary flow reserve; CI: confidence interval; CMR: cardiac magnetic resonance imaging; CV: cardiovascular; CYP2C19*2: CYP2C19 allele *2; CYP2C19*3: CYP2C19 allele *3; DAPT: dual antiplatelet therapy; DS: diameter stenosis; hs-CRP: high sensitivity C-reactive protein; HR: hazard ratio; IL-1ra: interleukin-1 receptor antagonist; IL-2: interleukin-2; IL-6: interleukin-6; IMR: index of microvascular resistance; LOF: loss-of-function; LVEDP: left ventricular end-diastolic pressure; LVEF: left ventricular ejection fraction; MACE: major adverse cardiovascular events; MI: myocardial infarction; MI-CAD: myocardial infarction with obstructive coronary artery disease; MINOCA: myocardial infarction with non-obstructive coronary arteries; MVO: microvascular obstruction; NSTEMI: non-ST-elevation myocardial infarction; NSTE-ACS: non-ST-elevation acute coronary syndrome; OCT: optical coherence tomography; PCI: percutaneous coronary intervention; PE: plaque erosion; 18F-FDG PET-CT: 2-Deoxy-2-[fluorine-18] fluoro-D-glucose positron emission tomography-computed tomography; PFT: platelet function tests; PICSO: pressure-controlled intermittent coronary sinus occlusion; pPCI: primary percutaneous coronary intervention; PR: plaque rupture; RRR: resistance reserve ratio; SAQSS: Seattle angina questionnaire summary score; s.c.: subcutaneous; STEMI: ST-elevation myocardial infarction; TIMI: thrombolysis in myocardial infarction; TLR: target lesion revascularization; TVR: target vessel revascularization.

### 2.2. Approach to Non-Culprit Lesions

The term “non-culprit lesions” refers to coronary plaques causing at least 50% stenosis that are not responsible for the index acute coronary event. As 40% of STEMI patients present multivessel coronary artery disease [59], management of non-culprit lesions is paramount [60]. The latest ACS guidelines from the European Society of Cardiology indicate a complete revascularization approach for STEMI patients with a class I recommendation, either during the index procedure or within 45 days [3]. This recommendation derives from the results of the COMPLETE (Complete Revascularization with Multivessel PCI for Myocardial Infarction) trial, which showed that among patients with STEMI and multivessel coronary artery disease performing complete revascularization is more effective than performing PCI on the culprit lesion alone in decreasing the risk of cardiovascular death or MI [61]. In a sub-study of the COMPLETE trial [62], OCT was performed on at least two coronary arteries before PCI on non-culprit lesions, showing that non-culprit obstructive plaques are more frequently TCFAs—or, in general, lipid-richer plaques—if compared with non-culprit non-obstructive plaques, and considering that approximately 50% of STEMI patients with multivessel coronary artery disease undergoing OCT evaluation had at least one obstructive vulnerable non-culprit plaque [62], the treatment with PCI of this obstructive vulnerable lesion may in part explain the clinical benefit derived from a complete revascularization in STEMI patients with multivessel obstructive disease [62]. The prognostic role of a vulnerable lesion has also been acknowledged in the PROSPECT (Providing Regional Observations to Study Predictors of Events in the Coronary Tree) II study, whereby a combination of near-infrared spectroscopy (NIRS) and IVUS was able to identify angiographically non-obstructive coronary artery lesions (vulnerable plaques) that were responsible for future adverse cardiac outcomes [63]. The highest-risk lesions had both a large plaque burden and high lipid content [63]. This hypothesis that unobstructive vulnerable plaque treatment with PCI may portend a favorable outcome has been evaluated by the PREVENT (Preventive percutaneous coronary intervention versus optimal medical therapy alone for the treatment of vulnerable atherosclerotic coronary plaques) trial [64]. Of interest, in this study preventive PCI on non-flow-limiting (fractional flow reserve > 0.80) lesions with high-risk features of vulnerability as assessed by intravascular imaging significantly reduced MACE if compared with optimal medical therapy alone [64]. In this study, plaque vulnerability was defined as the presence of at least two of the following characteristics on intracoronary imaging: minimal lumen area < 4 mm^2^, plaque burden > 70%; lipid-rich plaque on NIRS; and TCFA (lipid plaque with arc > 90° and fibrous cap thickness < 65 μm or a ≥ 10% confluent necrotic core with >30° abutting the lumen on OCT) [64]. However, this topic is still matter of debate and further evidence is needed before this approach can be recommended in clinical practice.

Regarding the timing of completion of revascularization, a recent meta-analysis evaluating studies that enrolled patients with NSTE-ACS and STEMI found no differences in terms of overall MACE at follow-up between staged and immediate revascularization, while immediate complete revascularization is possibly associated with a decreased risk of unplanned coronary revascularization and MI recurrence if compared with a staged approach [65,66].

### 2.3. Microvascular Obstruction Assessment

Another research field in which personalized diagnostic and therapeutic strategies could provide prognostic stratification and, in perspective, a clinical benefit to patients with STEMI is that of MVO (Table 1) [67]. MVO represents the pathophysiological mechanism underlying the no-reflow phenomenon: in a significant proportion of STEMI patients receiving primary PCI (pPCI) (variable from 10 to 67% depending on the adopted definition), restoration of epicardial coronary artery flow is not accompanied by adequate myocardial reperfusion, and the detection of MVO on cardiac magnetic resonance imaging (CMR) strongly predicts heart failure hospitalizations and mortality within 1 year [67,68]. Aside from the presence and extent of MVO on CMR, a further risk assessment after pPCI can be provided by microcirculatory functional invasive evaluation: higher zero-flow pressure (Pzf, defined as the distal coronary pressure at which point flow in a coronary artery would theoretically cease) has been shown to predict a larger MI extent on CMR at 6 months [69]. Furthermore, impaired coronary physiology indexes such as the coronary flow reserve (CFR) ≤ 1.25 and the index of microvascular resistance (IMR) > 31 units are associated with MVO detection on CMR and lower-left ventricular ejection fraction after STEMI [67]. Moreover, IMR measured at the end of pPCI may represent a clinically meaningful predictor of STEMI early cardiac complications, as patients with IMR ≤ 40 units have been shown to have an uneventful in-hospital recovery and may be safely observed in a lower-intensity environment [39].

In summary, an invasive coronary functional approach may provide a tailored risk estimation after primary PCI, allowing selection of patients who would benefit the most from future therapies aimed at restoring microvascular function, as well as providing a measurement of individual therapy response at follow-up [67,69]. A first example of IMR-guided adoption of a supplementary strategy for primary PCI in patients with a high likelihood of MVO has been provided by the OxAMI-PICSO (Oxford Acute Myocardial Infarction—Pressure-controlled Intermittent Coronary Sinus Occlusion) study: pressure-controlled intermittent coronary sinus occlusion (PICSO) in patients with anterior STEMI and a pre-stenting IMR > 40 units lowered IMR at 24–48 h and reduced infarct size at a 6-month follow-up [40]. However, the PiCSO-AMI-I (Pressure-controlled Intermittent Coronary Sinus Occlusion in Acute Myocardial Infarction) randomized trial did not provide evidence supporting a benefit from PICSO-assisted pPCI, as its use was not associated with a significant reduction of infarct size on CMR at 5 days, signaling that further investigations on approaches targeting MVO are required [41].

### 2.4. Antithrombotic Therapy

Considering pharmacological therapy DAPT with low-dose aspirin and a potent P2Y12 inhibitor (prasugrel or ticagrelor) represents the mainstay of antithrombotic therapy in ACS patients [13].

Given the variability of clinical settings, patient features, and individual drug response, personalized medicine plays a crucial role in determining a patient-tailored antithrombotic regimen. In fact, although a standard DAPT regimen is 12 months long, its duration can be adjusted: in high bleeding risk (HBR) patients, it can be shortened to as little as 1 month after ACS. Conversely, in non-HBR patients at high ischemic risk, it can be extended beyond 12 months. Regarding the choice of antiplatelet agents, clopidogrel is reserved for HBR patients or situations where a potent P2Y12 inhibitor is not available [70]. HBR criteria can be identified by a PRECISE-DAPT (Predicting Bleeding Complications In Patients Undergoing Stent Implantation and Subsequent Dual Anti Platelet Therapy) score of at least 25, a simple five-item risk score [71]. Another characterization has been provided by the Academic Research Consortium for High Bleeding Risk (ARC-HBR), with the objective to reach a consistent definition to be applied in clinical trials [72]. On the other hand, high ischemic risk can be defined by the presence of clinical risk factors and/or procedural technical complexity factors [70].

A suitable option for patients at high bleeding risk could be a de-escalation therapy, which consists in downgrading from a potent P2Y12 inhibitor at conventional doses to either clopidogrel or reduced-dose prasugrel [70,72,73]. In line with this, a recent randomized clinical trial, the TALOS-AMI (TicAgrelor Versus CLOpidogrel in Stabilized Patients With Acute Myocardial Infarction) study, showed that an unguided de-escalation strategy from ticagrelor-based to clopidogrel-based DAPT—in stabilized patients who experienced ACS and no major ischemic or bleeding events within the first month following index PCI—was clinically beneficial, due to a significant reduction in bleeding risk without a concomitant increase in ischemic risk [74]. On the other hand, the PEGASUS-TIMI 54 (Prevention of Cardiovascular Events in Patients with Prior Heart Attack Using Ticagrelor Compared to Placebo on a Background of Aspirin) trial demonstrated that prolonged ticagrelor-based DAPT decreased MACE in patients with a previous MI with or without preceding coronary stent implantation, at the expense of a higher bleeding risk [75].

Considering clopidogrel-based DAPT, the prevalence of individuals with high on-treatment platelet reactivity (HPR) is about 30% [70]. This observation derives from the nature of clopidogrel, which is a pro-drug and thus requires a double-step biotransformation process driven by the hepatic cytochrome P4502C19 enzyme [76]. HPR is a marker of inadequate clopidogrel-induced platelet inhibition and a predictor of recurrent thrombotic events, hindering de-escalation from potent P2Y12 inhibitors to clopidogrel in patients at high bleeding risk [70,77]. To evaluate the individual response to clopidogrel and guide de-escalation strategies, targeted tests can be carried out, including platelet function tests and genetic tests aiming at identifying loss-of-function alleles, carriers of the CYP2C19 gene (such as individuals who are carriers of alleles *2 and *3) [42,43,44,70,77]. Furthermore, diagnosing HPR through platelet function tests may allow a better understanding of the mechanisms underlying recent stent thrombosis in patients on P2Y12 inhibitor therapy and monitoring of medication adherence [77].

Conversely, a genotype-guided antiplatelet therapy escalation has been investigated by three randomized clinical trials (Table 1), two which showed a significantly lower rate of cardiovascular events in the pharmacogenomic arm [45,46], while risk reduction was non-significant in another study [47]. Overall, further evidence is needed in ordered to evaluate the clinical feasibility of a genotyping strategy in antiplatelet regimen escalation.

## 3. Precision Medicine in MINOCA

### 3.1. Diagnostic Process

MINOCA represents a “working diagnosis” that applies after exclusion of alternative overt causes for acute non-ischemic myocardial injury including cardiac (e.g., cardiomyopathies, cardiotoxicity, cardiac trauma, etc.) and extra-cardiac (e.g., pulmonary embolism, sepsis, stroke, etc.) conditions [3,78]. It is relevant to highlight that Takotsubo syndrome or acute myocarditis are not classified as MINOCA anymore but are now considered non-ischemic myocardial infarction mimics [78,79].

**Figure 2:**
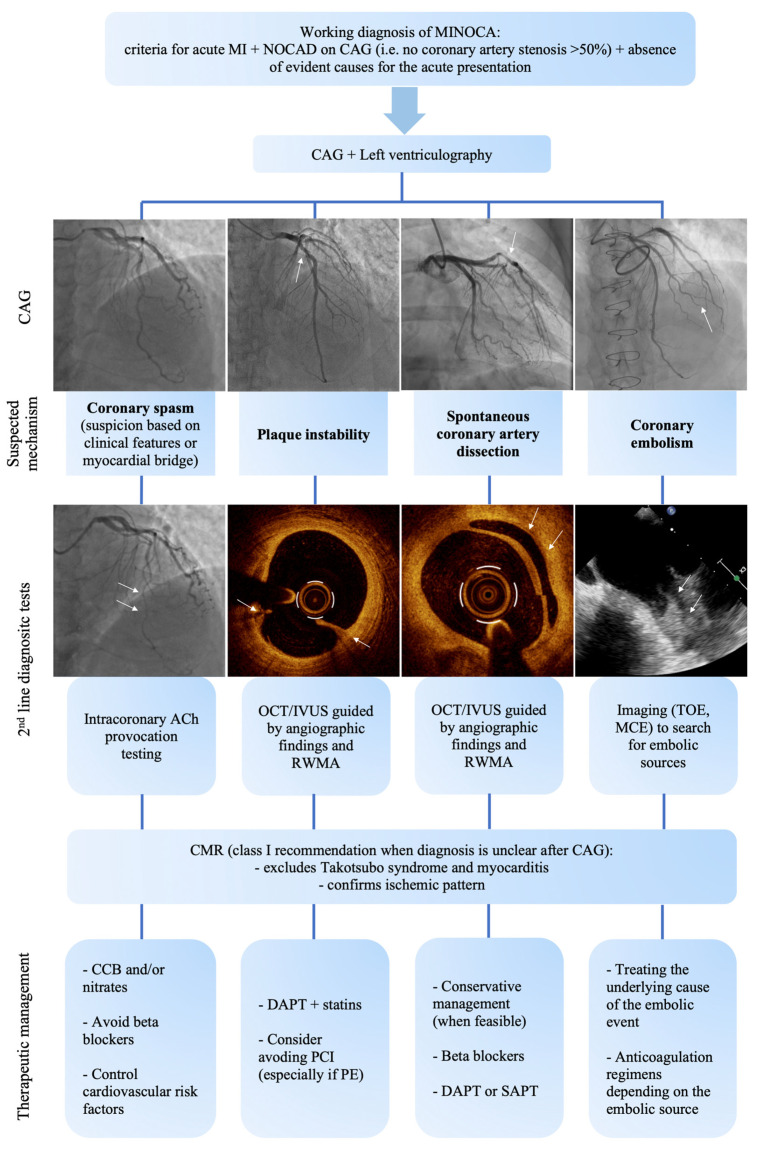
Tailored diagnostic and therapeutic approach in myocardial infarction with non-obstructive coronary arteries. Abbreviations: ACh: acetylcholine; CAG: coronary angiography; CCB: calcium channel blocker; CMR: cardiac magnetic resonance imaging; DAPT: dual antiplatelet therapy; IVUS: intravascular ultrasound; MCE: myocardial contrast echocardiography; MI: myocardial infarction; MINOCA: myocardial infarction with non-obstructive coronary arteries; NOCAD: non-obstructive coronary artery disease; OCT: optical coherence tomography; PE: plaque erosion; RWMA: regional wall-motion abnormalities; SAPT: single antiplatelet therapy; TOE: transesophageal echocardiography.

Managing patients with MINOCA requires a tailored multimodal diagnostic approach to rule out the aforementioned non-ischemic causal hypotheses and identify the specific mechanism leading to MI, allowing an appropriate personalized therapy (Figure 2) [6]. In fact, pathophysiologic pathways underlying MINOCA are diverse and include coronary artery spasm, plaque instability (due to PR or PE), coronary embolism, and non-obstructive SCAD [78,80]. A prolonged vasospasm at an epicardial or microvascular level, caused by vascular smooth muscle cell hyperreactivity and favored by endothelial dysfunction, can result in MI [81]. A destabilized non-obstructive plaque can be responsible for MINOCA when it leads to transient thrombotic coronary obstruction, potentially accompanied by distal thrombus embolization and coronary spasm due to the local release of vasoactive mediators [82]. Patients with positive remodeling of atherosclerotic coronary arteries and efficient endogenous thrombolysis may be particularly predisposed to this mechanism [83]. Embolic material arising from thrombi, valves, or neoplasms can lead to coronary embolism [84]. SCAD is caused by the development of a hematoma within the tunica media, which separates the intima from the underlying vessel leading to dynamic lumen compression [85].

Coronary angiography is the gatekeeper diagnostic exam in patients presenting with an ACS [3], and 2023 European guidelines suggest left ventriculography, functional coronary angiography, and intravascular imaging as additional instruments to establish a definite diagnosis in MINOCA [3].

Coronary angiography itself may reveal hazy lesions, limited contrast defects, or wall irregularities raising the suspicion of plaque instability [6,82]. Furthermore, non-obstructive SCAD may be angiographically suspected in the presence of gradual vessel tapering [78], while coronary embolism may result in abrupt filling defects of distal branches in multiple coronary territories, residual thrombotic material in epicardial arteries, and/or a TIMI flow < 3 [84,86]. Intracoronary imaging with IVUS or OCT allows confirmation of a diagnosis of SCAD, to rule out ruptured or eroded culprit plaques, especially in presence of ambiguous non-obstructive lesions [12,48,82]. The choice of coronary vessels to be investigated through intracoronary imaging is guided not only by angiographic findings but also by regional wall-motion abnormalities on transthoracic echocardiography or ventriculography and/or ECG features [12].

Intracoronary acetylcholine (ACh) provocative testing allows the diagnosis of coronary epicardial or microvascular spasm and has been proven safe in the acute setting [81,87]. A positive ACh test has prognostic relevance in patients with MINOCA, being associated with a higher incidence of cardiovascular events [81,88]. Interestingly, ACh testing is more frequently positive in patients with myocardial bridging, an association described in about 20% of MINOCA patients with suspected vasomotor disorders and related to worse outcome [89,90]. Recently, the ABCD score, which includes factors such as acute presentation as MINOCA, the presence of a myocardial bridge, elevated hs-CRP levels, and dyslipidemia, has been demonstrated to accurately predict positivity in ACh testing [90]. Accordingly, its use might avoid performing ACh testing in selected subsets of patients or reaching a diagnosis whereby an ACh test is not available [88,90].

CMR is of particular relevance in a MINOCA diagnostic work-up: according to the latest ACS European guidelines, CMR is indicated with a class I level B recommendation in MINOCA patients with an unclear diagnosis after coronary angiography [3]. T2-weighted sequences detect acute damage-related myocardial edema or inflammation, while late gadolinium enhancement differentiates ischemic patterns of myocardial scarring/fibrosis (transmural, subendocardial, or focal) from those consistent with a non-ischemic process (mid-wall or epicardial) associated with Takotsubo syndrome or myocarditis [86,91]. However, the absence of scarring on CMR does not exclude a MINOCA diagnosis, as troponin elevation may express an aborted permanent myocardial lesion due to spontaneous coronary recanalization [91].

Transthoracic echocardiography (TTE) plays a fundamental role since the first diagnostic steps in MINOCA patients, revealing potential embolic sources [6,86] such as prosthetic heart valves, infective endocarditis, cardiac neoplasms, patent foramen ovale/interatrial defect [92], and left ventricular apical thrombus [93]. For the latter two, integration with echo-contrast agents may be helpful [6,86]. Furthermore, regional wall-motion abnormality distribution on TTE provides information on the possible pathogenetic mechanism of myocardial injury, suggesting PR, SCAD, or epicardial spasm when affecting the distribution territory of a single epicardial coronary artery, while diffuse abnormalities are more consistent with microvascular spasm, myocarditis, or Takotsubo syndrome [86]. A tailored diagnosis may be further supported by myocardial contrast echocardiography (MCE), which enables differentiation between epicardial and microvascular patterns of myocardial perfusion defects [86]. Transesophageal echocardiography (TOE) allows better evaluation of mechanical valves or intracavitary masses and the diagnosis of auricular thrombosis [94].

### 3.2. Tailored Therapy in MINOCA

To date, no clinical guideline addresses personalized therapeutic strategies in patients with MINOCA, given the paucity of randomized data and the variety of mechanisms involved [6,48]. However, a diagnostic suspicion confirmed by targeted tests may guide treatment and is recommended by clinical guidelines (Figure 2) [3,78]. Among patients with MINOCA due to epicardial or microvascular coronary spasm (established by invasive provocative testing), calcium channel blockers (CCBs) represent the first-line treatment to improve both symptoms and prognosis, and CCB dose reduction or discontinuation at follow-up has been associated with a higher rate of mortality [81]. Furthermore, considering MINOCA patients with evidence of myocardial bridging and positive ACh testing, β-blockers should be avoided because they may favor coronary spasm by unmasking α-adrenoreceptors, while CCBs should be preferred [95].

When plaque instability is confirmed or suspected as the pathogenetic mechanism, statin therapy together with 1 year of DAPT followed by single antiplatelet therapy (SAPT) is the recommended approach, even in the presence of mild atherosclerosis, while stent implantation should be considered in each single case according to lesion characteristics [5]. In patients in whom non-obstructive plaque erosion has been identified, a conservative antiplatelet therapy with aspirin and ticagrelor may be considered, and it has been proven to be effective in terms of MACE prevention, avoiding the risk of stent-related complications [22,37,38].

Conservative management is also advised in patients with SCAD, whenever possible [3]: PCI should be reserved for patients with left main involvement, ongoing ischemia, or hemodynamic instability, given the high complication rate of revascularization [85,96]. Antiplatelet therapy in patients with SCAD not undergoing PCI is currently a matter of debate. Indeed, despite previous consensus on the importance of the DAPT regimen in this context [85], the recent evidence from the DISCO study demonstrated a higher rate of MACE in patients with SAPT compared to DAPT [97].

In case of coronary embolism, treatment should be further personalized depending on the probable embolic source, considering that antiplatelet therapy may be indicated when coronary thrombosis with subsequent embolism is suspected, while different anticoagulation regimens are the treatment of choice in systemic or venous paradoxical embolism, depending on the etiology and presence of other embolic sites [78,84,98].

Due to the heterogeneous mechanisms underlying MINOCA, it is clear that the use of DAPT should be restricted to patients effectively requiring this approach. Indeed, the SWEDEHEART registry clearly demonstrated no benefit of DAPT use in an unselected cohort of patients presenting with MINOCA [99,100].

### 3.3. Future Perspectives in MINOCA-Tailored Therapy

To date, no published randomized trial has evaluated personalized diagnostic and therapeutic approaches in etiology-stratified MINOCA patients. Signals coming from observational data suggest a beneficial effect of angiotensin-converting enzyme inhibitors (ACEIs)/angiotensin receptor blockers (ARBs) and statins in MINOCA prognosis, with a non-significant effect of β-blockers, CCBs, and antiplatelet agents [100,101,102], though these data were obtained in cohorts of patients who were not differentiated on a pathophysiological basis. The PROMISE (Prognostic Value of Precision Medicine in Patients With MINOCA) trial is going to evaluate the implications on prognosis, quality of life, and healthcare costs of tailored management (including a personalized diagnostic pathway and a consequent stratified treatment) in MINOCA patients randomized to either a standard or precision approach (ClinicalTrials.gov: NCT05122780) [48]. The results coming from this trial will hopefully strengthen the indication for a targeted treatment of MINOCA based on pathophysiological mechanisms identified through a comprehensive work-up (Table 1).

Furthermore, StratMed-MINOCA (Stratified Medicine of Eplerenone in Acute MI/Injury) is an ongoing clinical trial evaluating eplerenone ability to reduce myocardial damage (indicated by NT-proBNP) in MINOCA patients with coronary microvascular dysfunction (CMD) as assessed by the index of microvascular resistance (IMR) ≥ 25 (ClinicalTrials.gov Identifier: NCT05198791) [49].

## 4. Tailored Prevention Approaches

### 4.1. Inflammatory Markers and Anti-Inflammatory Therapies

An increase of inflammatory markers—defined by >25% change in circulating concentration during an inflammatory response—in ACS is an already well-known association [103]. It has been already demonstrated that the elevation of sensitive acute-phase proteins such as C-reactive protein (CRP) and serum amyloid A protein at hospital admission for ACS predicts a poor outcome [30]. Several inflammatory markers, such as myeloperoxidase, lipoprotein-associated phospholipase A2 (Lp-PLA2), pentraxin-3, interleukin-6 (IL-6), tumor necrosis factor (TNF)-alpha, matrix metalloproteinase 9, and CRP are involved in the pathophysiology of atherosclerotic vascular disease [104]. Among these biomarkers, CRP has become one of the most useful in clinical practice due to the availability of high-sensitivity assays (hs-CRP) [105]. As underscored by the PROVE-IT TIMI 22 (Pravastatin or Atorvastatin Evaluation and Infection Therapy-Thrombolysis In Myocardial Infarction 22) trial, lowering LDL cholesterol levels with statin therapy following an ACS allowed the most favorable clinical outcomes in patients who not only achieved LDL cholesterol concentration below 70 mg/dL but also attained hs-CRP levels below 2 mg/L [106]. This observation is in line with the results of the JUPITER (Justification for the Use of statins in Prevention: an Intervention Trial Evaluating Rosuvastatin) trial, which showed that rosuvastatin caused a 44% MACE reduction in healthy patients without hyperlipidemia but with elevated levels of hs-CRP [107]. These studies confirm the role of statins as effective anti-inflammatory drugs, both successful in lowering LDL cholesterol levels and in mitigating inflammatory processes. Results from the IMPROVE-IT (IMProved Reduction of Outcomes: Vytorin Efficacy International Trial) trial, adopting the addition of ezetimibe to simvastatin, further confirmed the relevance of reducing hs-CRP levels aside from LDL cholesterol to provide a benefit in terms of cardiovascular events in ACS survivors, and suggested that reaching cholesterol and CRP targets may be more important per se than the mechanism of achievement [108]. Considering other non-statin-based therapeutic strategies, and in particular proprotein convertase subtilisin/kexin type 9 (PCSK9) inhibitors, research has provided conflicting results regarding residual inflammatory risk reduction, as bococizumab showed similar relative risk reductions for cardiovascular events among various hs-CRP strata [109], while evolocumab provided a greater absolute benefit in patients with higher hs-CRP at baseline [110].

With regard to targeted inflammatory pathway inhibition (Table 1), the MRC-ILA (Medical Royal Council InterLeukin-1 Antagonist) Heart Study showed that patients diagnosed with NSTE-ACS and assigned to receive anakinra, a recombinant interleukin-1 (IL-1) receptor antagonist, manifested a consistent reduction of CRP levels during the first 7 days after ACS, compared to patients assigned to the placebo group [51]. On the other hand, the anakinra-treated group experienced an excess in MACE at a 1-year follow-up, a signal to be better understood in future studies [51]. This trial highlighted how blocking IL-1 could mitigate the acute inflammatory response observed in ACS and demonstrated that IL-1 plays a pivotal role as a mediator in the systemic inflammatory response in ACS [51]. This observation has been confirmed by the CANTOS (Canakinumab Anti-inflammatory Thrombosis Outcome Study), which brought the striking result that patients with previous MI treated with canakinumab, a monoclonal antibody targeting the interleukin (IL)-1β innate immunity pathway, at a dosage of 150 mg every 3 months, showed a significantly reduced incidence of recurrent cardiovascular events compared to placebo [50]. The potential benefit of anakinra treatment in ACS was later explored in STEMI patients by Abbate et al. in a phase 2 trial [52]: a strong reduction in hs-CRP was observed in the treated arm, together with improved heart failure outcomes. Furthermore, the risk for ischemic recurrences was not increased in patients receiving anakinra in this trial, different from the MRC-ILA Heart Study [51,52]. Another targeted approach has been tested with IL-6 inhibition: in STEMI patients, tocilizumab has been shown to increase myocardial salvage index as assessed by CMR and to reduce MVO, although no difference in infarct size was observed if compared with placebo [53]. Furthermore, low-dose interleukin (IL)-2, which enhances regulatory T-cell expression, thus limiting chronic inflammation, has been proved to be safe and biologically effective in NSTE-ACS patients by the LILACS (Low-dose InterLeukin 2 in patients with stable ischemic heart disease and Acute Coronary Syndromes) study [54]. IL-2 efficacy is being further investigated in the context of ACS by the IVORY (Low-dose interleukin 2 for the reduction of vascular inflammation in acute coronary syndromes) trial (NCT04241601), with index vessel inflammatory parameters as assessed by PET-CT scan as the primary endpoint [55].

In contrast, an alternative approach targeting inflammatory processes with low-dose methotrexate did not influence inflammatory marker levels (i.e., IL-1β, IL-6, and hs-CRP) or cardiovascular outcomes in patients with previous MI or multivessel coronary artery disease but resulted in an increased adverse event rate in the treated arm [111]. On the other hand, the COLCOT (Colchicine Cardiovascular Outcomes Trial) provided promising results regarding treatment with colchicine at 0.5 mg daily in patients with recent MI, as it was associated with a significantly lower occurrence of ischemic cardiovascular events compared to placebo, without increasing drug-related adverse events [56], a benefit in MACE reduction that was later confirmed in patients with stable coronary artery disease by the LoDoCo2 (Low-Dose Colchicine 2) trial [112]. Colchicine may be beneficial on atherosclerosis mechanisms due to its inhibition of the NLR family pyrin domain containing 3 (NLPR3) inflammasome, which leads to a downregulation of the IL-1β/IL-6/hs-CRP pathway [113]. The efficacy of colchicine in the secondary prevention of ACS is being further evaluated by the ongoing CLEAR-SYNERGY (Colchicine and Spironolactone in Patients With MI/SYNERGY Stent Registry) trial (NCT03048825) in combination with spironolactone in a 2 × 2 factorial design, which may clarify colchicine’s role in reducing MACE at follow-up [57].

Lastly, leukotrienes represent pro-inflammatory vasoconstrictive agents, and inhibition of their biosynthesis has been recently evaluated by the FLAVOUR (phase IIa efficacy study of the 5-lipoxygenase activating protein antagonist AZD5718 in patients with recent myocardial infarction) study in patients with recent MI: urinary leukotriene levels were significantly reduced, while no effect on non-invasive CFR (the key secondary endpoint) was observed [58].

In conclusion, an inflammatory burden reduction strategy in ACS may represent an effective approach both for primary and for secondary prevention, but further studies are required to allow its inclusion in standard clinical practice. In fact, the pathways studied as therapeutic targets by previous trials are various, and the endpoints considered are heterogeneous, while data are currently lacking on differential effects depending on the type of ACS and regarding the best timing for anti-inflammatory therapy administration.

### 4.2. Beyond Traditional Risk Factors: Considering the Role of the Exposome

In recent years, the concept of “exposome” has gained attention in cardiovascular medicine: “non-traditional” risk factors including air pollution, light pollution, noise pollution, social stress, and infectious diseases interact in non-linear ways increasing the risk of ischemic heart disease [114]. Among these, air pollution is the most known and studied factor, considering its relevance in the pathogenesis of several systemic and cardiovascular diseases: about 20% of cardiovascular deaths are attributable to outdoor and indoor air pollutants [115,116]. An association is described between increased levels of pollutants—primarily particulate matter < 2.5 μm (PM2.5)—and acute coronary ischemic events due to both plaque instability and vasomotor abnormalities [114,117,118,119,120]. In addition, in patients with previous ACS survival is negatively affected by long-term PM2.5 exposure [121,122]. This evidence suggests how pollution-mitigating strategies—both governmental and personal—could be highly effective in reducing air pollutant-mediated cardiovascular risk: shifting to low-emission vehicle mobility and to renewable energy sources and adopting home air-cleaning devices, personal protective equipment including FFP2 face masks, and air purifiers [114]. It has been shown that the use of portable or installed air purifiers improves various indicators of cardiovascular health, including blood pressure, insulin sensitivity, inflammatory markers, stress hormones, and metabolomic profiles [121,123].

## 5. Conclusions

A precision medicine strategy by means of invasive and non-invasive complementary diagnostic tests and targeted therapies in ACS is being increasingly recognized as being impactful on patients’ prognosis, both in the context of MI-CAD and MINOCA. Intravascular imaging and functional testing provide guidance for interventional approaches for culprit and non-culprit plaques, while inflammatory biomarkers contribute to an etiological stratification of ACS and suggest pathophysiological pathways to target in the future. In fact, OCT-guided MI-CAD endotype assessment predicts long-term clinical outcome and portends relevant therapeutic implications, including the possibility of stent-free management in selected cases. Furthermore, invasive coronary physiological evaluation provides future perspectives in MVO assessment and treatment. MINOCA patients may benefit from a comprehensive approach, including coronary angiography, intravascular imaging, ACh provocative testing, TTE, CMR, and TEE, as needed. Therapeutic implications for each MINOCA subtype are distinct, though validation by randomized trial is needed.

In conclusion, “precision medicine” in ACS may confer further prognostic benefits, optimizing therapeutic resources in patients undergoing a multimodality diagnostic pathway and minimizing the residual risk.
